# Effect of upper eyelid blepharoplasty on the ocular surface, tear film, and corneal microstructure

**DOI:** 10.5935/0004-2749.2022-0220

**Published:** 2024-03-05

**Authors:** Orhan Aygun, Yonca Ozkan Arat, Ozlem Dikmetas, Jale Karakaya, Ata Baytaroglu, Murat Irkec

**Affiliations:** 1 Department of Ophthalmology, Faculty of Medicine, Yeni Yüzyıl University, Istanbul, Turkey; 2 Department of Ophthalmology, Faculty of Medicine, Hacettepe University, Ankara, Turkey; 3 Department of Biostatistics, Faculty of Medicine, Hacettepe University, Ankara, Turkey; 4 Department of Ophthalmology, Usak Training and Research Hospital, Usak, Turkey

**Keywords:** Blepharoplasty, Intravital microscopy, Lissamine green dyes, Fluorescein, Severity of illness index, Dry eye syndromes

## Abstract

**Purpose:**

This study aimed to investigate the effect of upper eyelid blepharoplasty with the removal of the skin and a strip of orbicularis oculi muscle on the ocular surface, tear film, and dry eye-related symptoms.

**Methods:**

Twenty-two eyes of 22 consecutive patients operated by a single surgeon (21 females; mean age, 61 years; age range, 41-75 years) were included. All subjects completed the Ocular Surface Disease Index questionnaire, underwent *in vivo* confocal microscopy, tear film breakup time measurements, the Schirmer test with anesthesia, and fluorescein and lissamine green staining measurements before, 1 month, and 6 months after upper blepharoplasty alone with preseptal orbicularis excision.

**Results:**

A significant increase in Ocular Surface Disease Index, and corneal fluorescein and lissamine green staining and a significant decrease in tear film breakup time were observed after 1 month (p=0.003, p=0.004, p=0.029, and p=0.024 respectively) and 6 months (p=0.001 for all findings). No significant difference in the Schirmer test score was observed during the follow-up. None of the *in vivo* confocal microscopy parameters showed significant changes during the study.

**Conclusions:**

An increase in dry eye symptoms and a decrease in tear film stability along with ocular surface staining were observed in patients undergoing upper eyelid blepharoplasty.

## INTRODUCTION

Upper eyelid blepharoplasty improves patients’ quality of life by improving visual function and esthetic outcome^(1)^. Several retrospective studies, most of which included both upper and lower eyelid blepharoplasties, have reported increased dry eye complaints following blepharoplasty^(2,3,4)^. The concept that upper eyelid blepharoplasty alone with skin and orbicularis excision leads to dry eye symptoms has not been clearly confirmed by prospective studies in the literature. As a matter of fact, of the five prospective studies that included upper eyelid blepharoplasties with skin and orbicularis excision only, three suggested the contrary^(5,6,7)^, as opposed to the two remaining studies^(8,9)^. One recent prospective study involving subjects with and without preexisting dry eye symptoms found that the release of tear inflammatory cytokines and tear film instability that occur after upper blepharoplasty in the early postoperative period mostly resolved after 6 months in healthy subjects, whereas worse and persistent ocular surface damage was noted in subjects with preexisting dry eyes^(10)^. Because of the conflicting data in the literature on this subject, we aimed to clarify the effects of upper eyelid blepharoplasty with skin and preseptal orbicularis excision on the ocular surface and tear film.

*In vivo* confocal microscopy (IVCM) allows the evaluation of the signs and symptoms of ocular surface damage in dry eye disease at a cellular level^(11,12)^. In this study, we aimed to evaluate dry eye-related ocular surface damage after upper eyelid blepharoplasty at a cellular level by performing IVCM.

## METHODS

The study was approved by the Clinical Research Ethics Board of (redacted) University, and informed consent was obtained from all patients before study initiation. This study was conducted according to the tenets of the Declaration of Helsinki.

This was a prospective study involving 22 eyes of 22 consecutive patients presenting to our clinic between April 2016 and March 2019 who underwent upper eyelid blepharoplasty by a single surgeon (YOA). The right eyes of the patients were evaluated using dry eye tests and IVCM. Patients with ocular and eyelid pathology/ malposition; Sjogren syndrome; dry eye disease; systemic diseases that would affect the ocular surface, including rheumatoid arthritis, scleroderma, and systemic lupus erythematosus; Stevens-Johnson syndrome; and a history of previous eyelid surgery were excluded. Moreover, those who used contact lenses were also excluded. All subjects completed the Ocular Surface Disea se Index (OSDI) questionnaire. All patients underwent comprehensive ophthalmic examination, including an ocular surface assessment, tear film breakup time (TBUT), Schirmer test with anesthesia, and fluorescein and lissamine green staining measurements, along with IVCM (ConfoScan 4 confocal microscope, Nidek, Inc., Fremont, CA), at baseline, 1 month, and 6 months after surgery. TBUT, fluorescein and lissamine green staining measurements, and Schirmer test were performed sequentially by the same ophthalmologist (OA) The tests were performed in the same order on each patient. IVCM was performed last by the same ophthalmologist (OD) on each patient. The temperature and humidity of the examination room during all tests were maintained at 25°C and 30%, respectively. TBUT was measured by wetting a fluorescein strip with saline and then instilling a uniform drop of fluorescein in the inferior fornix. The patient was instructed to blink three times and then to stop blinking. The time was recorded when the first black spot was observed on the corneal surface under a slit lamp using cobalt blue light. Then, the ocular surface was observed and graded for fluorescein staining^(13)^. Similarly, a lissamine green strip was wet with saline, and the whole drop retained on the strip was instilled inside the far lower temporal lid in upgaze^(13)^. Staining with both stains was graded using the Oxford Scheme 6-point scale. The intensity of fluorescein and lissamine green staining was graded using the 6-point scale, ranging from 0 (lowest) to 5 (highest), according to the Oxford grading scheme^(14)^. The Schirmer test with anesthesia was performed by folding the Schirmer paper strip (5 × 35 mm) at the notch and hooking the folded end over the temporal one-third of the lower lid margin after instillation of 1 drop of proparacaine hydrochloride 0.5% and light blotting of residual fluid in the inferior fornix^(13)^. Only the examination and measurements of the right eye were evaluated.

All subjects underwent standard bilateral upper eyelid blepharoplasty under local anesthesia involving skin and partial preseptal orbicularis excision without fat removal, which was performed by a single oculoplastic surgeon. At least 20 mm of residual skin was left between the brow and eyelid margin in all cases. After placing skin markings, a skin incision was performed using a no.15 blade. Excess eyelid skin and a partial strip of the preseptal orbicularis muscle were excised bilaterally. Bipolar cautery was used for hemostasis. The skin was closed using a 6-0 polyglactin 910 suture in an interrupted fashion. The patients were instructed to apply cold compresses for 24 h after surgery and to use an ointment containing tobramycin (0.3%) and dexamethasone (% 0.1) two times per day for 1 week along the incision site.

IVCM was used to measure basal epithelial cell density, anterior and posterior keratocyte cell density, the number of long nerve fibers, corneal sub-basal nerve density, and nerve tortuosity. The noncontact mode of ConfoScan 4 attached to a noncontact 20**×** lens on the central corneas of all subjects was used to obtain nerve measurements. The calculations were performed using Neuron J (http://www.imagescience.org/meijering/software/neuron). The number of long nerve fibers was calculated per frame. Sub-basal nerve tortuosity and the number of long nerve fibers were measured by an observer who was blinded to the underlying diagnoses of the subjects under study^(15,16)^.

### Statistical methods

Numerical variables were evaluated for the normality of data distribution using the Kolmogorov-Smirnov test. Descriptive statistics are expressed as means ± standard deviations according to the assumption of a normal distribution. One-way repeated-measures analysis of variance (ANOVA) was used to assess the significance of change within times for variables. After one-way repeated-measures ANOVA, a post hoc Bonferroni test was used for multiple comparisons. Friedman’s test was used to evaluate the significance of differences over time for nonnormal quantitative variables. P-values <0.05 were used to denote statistical significance. All statistical analyses were performed using Statistical Package for the Social Sciences, version 23.0. Power analysis was performed using the variables presented in table 1 that comprised the primary aim of the study. The observed power of the study ranged from 0.64 to 0.95 for the different variables (0.95 for fluorescein staining measurements, 0.95 for lissamine green staining measurements, 0.80 for TBUT, 0.64 for the Schirmer test, and 0.76 for the OSDI).

**Table 1:** Ocular surface disease index scores and ocular surface parameters before and after upper eyelid blepharoplasty

	Baseline mean ± SD	1 month mean ± SD	6 months mean ± SD	p-value
OSDI	30.65 ± 18.27	35.05 ± 19.68	40.97 ± 24.06	<0.000
Fluorescein staining	0.50 ± 0.59	1.13 ± 0.63	1.63 ± 0.72	<0.000
Lissamine green staining	0.63 ± 0.58	1.13 ± 0.56	1.63 ± 0.72	<0.000
TBUT (seconds)	7.50 ± 3.24	6.23 ± 2.79	5.50 ± 3.86	<0.000
Schirmer test with anesthesia (mm/5 min)	13.86 ± 4.55	12.22 ± 4.62	11.86 ± 4.70	0.31

SD= standard deviation; OSDI= Ocular Surface Disease Index; TBUT= tear breakup time.

## RESULTS

Twenty-two patients (21 female and 1 male) with a mean age of 61 years (range: 41-75 years) who underwent upper eyelid blepharoplasty were included in the study. No complications, including lagophthalmos, were noted after surgery. All patients had a satisfactory outcome. All patients completed the entire follow-up.

A significant increase in the mean OSDI scores was observed at 1 month (35.05 ± 19.68; p=0.003) (median: 27.1) and 6 months (40.97 ± 24.06; p=0.001) (median: 37.5) compared with those at baseline (30.65 ± 18.27)(median: 28.1) (Table 1). Moreover, a significant increase in the mean corneal fluorescein and lissamine green staining scores was observed 1 month (1.13 ± 0.63, p=0.004, and 1.13 ± 0.56, p=0.029, respectively) and 6 months (1.63 ± 0.72, p=0.001 and 1.63 ± 0.72, p=0.001, respectively) after upper eyelid blepharoplasty compared with those at baseline (0.50 ± 0.59 and 0.63 ± 0.58, respectively) (Table 1). A significant decrease in the mean TBUT was observed 1 month (6.23 ± 2.79 s, p=0.024) and 6 months (5.50 ± 3.86 s, p=0.001) after upper eyelid blepharoplasty compared with preoperative values (7.50 ± 3.24 s). No significant changes in the Schirmer test scores were observed 1 and 6 months after surgery compared with those at baseline (p=0.31) (Table 1).

None of the IVCM parameters, including basal epithelial cell density (p=0.48), anterior and posterior keratocyte density (p=0.53 and p=0.81 respectively), total sub-basal nerve fiber density (p=0.52), the number of long sub-basal nerve fibers (p=0.59), and nerve tortuo sity (p=0.36), showed a significant change after surgery compared with preoperative values (Table 2).

**Table 2:** In vivo confocal microscopic fndings before and after upper eyelid blepharoplasty

	Baseline mean ± SD	1 month mean ± SD	6 months mean ± SD	p-value
Basal epithelial cell density (cells/)	3745.31 (± 350.59)	3743.50 (± 347.10)	3782.55 (±368.46)	0.48
Anterior keratocyte cells density (cells/)	609.86 (± 75.5)	618.45 (± 84.77)	618.86 (±83.50)	0.53
Posterior keratocyte cells density (cells/)	486.82 (± 76.30)	479.18 (± 84.84)	495.32 (±86.95)	0.81
Mean total subbasal nerve fiber density (µm/frame)	2129.97 (± 68.23)	2126 (± 68.68)	2126 (±68.68)	0.52
Long subbasal nerve branch number (nerves/frame)	8.00 (6-12)	9.00 (5-11)	8.00 (6-12)	0.59
Nerve Tortuosity	1.00 (1-2)	1.00 (1-2)	1.00 (1-2)	0.36

## DISCUSSION

According to the TFOS DEWS II Iatrogenic Dry Eye Subcommittee, dry eye disease may be a consequence of several iatrogenic interventions, including topical or systemic medications, contact lens use, ophthalmic surgery, and nonsurgical procedures^(17,18)^. Keratoplasty, eyelid surgeries, and cataract and refractive surgery are the most common ophthalmic interventions that may lead to dry eye disease^(17)^.

Eyelid surgery that causes the onset of dry eye disease or worsening of preoperative dry eye complaints is thought to be common but underreported^(19)^. The incidence of dry eye symptoms following cosmetic blepharoplasty has been reported to be 0%-26.5%^(3,20)^. According to a 10-year single-center retrospective study, of 892 patients undergoing blepharoplasty, 26.5% reported dry eye symptoms at some point after surgery^(3)^. Among them, combined upper and lower eyelid blepharoplasty (31.3%) was more likely to cause dry eye symptoms than upper eyelid blepharoplasty (12.9%) and lower eyelid blepharoplasty alone (21.4%). In a study involving a survey of ophthalmic plastic surgeons with expertise in blepharoplasty surgery, 77.5% of respondents reported dry eye complaints in the first 30 days (0%-20%). They noted that 0%-73% of patients continued to have persistent dry eye after the initial 30 days following surgery, mostly in the 0%-10% range^(21)^. Although some studies suggested a causal relationship between dry eye disease and blepharoplasty^(2,3,4,22)^, they are limited in estimating the true incidence of dry eye disease after upper eyelid blepharoplasty because of their retrospective design, nonstandard surgery technique, the inclusion of both upper and lower eyelid blepharoplasties with or without additional procedures, or the use of mostly patient-reported symptomatology for diagnosing dry eye disease rather than supporting it with ocular surface findings and comprehensive tear function tests.

In this study, which included patients undergoing upper eyelid blepharoplasty with skin and partial orbicularis excision, we noted a significant increase in the OSDI and corneal fluorescein and lissamine green staining scores and a significant decrease in TBUT after surgery compared with those at baseline. The Schirmer test score did not differ statistically during the follow-up. This finding suggests that aqueous layer deficiency is unlikely to cause dry eye disease following upper eyelid blepharoplasty as supported by previous studies in the literature^(6,8)^. None of the IVCM parameters showed a significant change in this study.

Standard upper eyelid blepharoplasty includes the removal of both the excess skin and a part of the underlying orbicularis muscle with or without the removal of fat^(23)^. Despite the changes in the surgical technique over the years toward more conservative approaches, including muscle-sparing blepharoplasty, a recent study involving a survey among Aesthetic Society members regarding the upper eyelid blepharoplasty practice patterns revealed that most surgeons (44.8%) perform a combination of skin, muscle, and orbital fat manipulation depending on the presenting features of the patient. Only 10% of the respondents reported that their most common technique involved skin excision only and 18.4% reported skin excision with muscle preservation and orbital fat manipulation^(24)^. In addition to providing volume restoration in some patients, the idea behind muscle preservation is to possibly decrease the incidence or progression of dry eye disease^(24)^. Even though upper eyelid blepharoplasty, particularly with the removal of a strip of the orbicularis oculi muscle, is thought to change the eyelid closure and tear pumping dynamics and can decrease the blink rate^(4,17,25)^, this hypothesis has not been confirmed by two prospective studies that showed that eyelid dynamics represented by blink frequency and an incomplete blink rate were not significantly changed after upper eyelid blepharoplasty^(8,10)^.

Only five prospective studies investigated dry eye disease following upper eyelid blepharoplasty with orbicularis excision; however, these study reported conflicting results^(5,6,7,8,10,)^. Mak et al. reported an increase in dry eye symptoms in six of seven patients with dermatochalasis undergoing upper eyelid blepharoplasty involving the removal of the skin and a strip of the orbicularis oculi with a significant increase in OSDI scores and no significant changes in the Schirmer test and TBUT^(8)^. Another study involving 14 patients looking at only TBUT using Keratograph 5M following upper eyelid blepharoplasty with the removal of the skin and a small amount of the preseptal orbicularis reported no significant changes in TBUT^(5)^. However, both studies have similar limitations: small number of cases and the inclusion of both eyes of the same subject in the evaluation. One recent prospective study involving 54 healthy subjects showed that dry eye-related symptoms were reduced and that tear dynamics were unaffected after upper blepharoplasty with or without the removal of a strip of the orbicularis muscle^(7)^. Our results are not in agreement with the findings of this study. We believe that the main reason for this disagreement is that our patient group had much higher preoperative OSDI scores (median: 28.1) than those in that study (13 in the first group and 17 in the second group). Patients with preexisting subjective dry eye complaints might be more prone to an increase in dry eye symptoms and a decrease in tear film stability after blepharoplasty. This hypothesis is further supported by another recent study comparing healthy subjects with subjects with a preexistent dry eye disease. In that study, the values of noninvasive TBUT and fluorescein TBUT in subjects with preexisting dry eye disease significantly decreased 1, 3, and 6 months after surgery compared with the preoperative measurements. Furthermore, interleukin (IL)-6 and IL-8 remained higher than baseline after upper eyelid blepharoplasty with orbicularis excision in this group of subjects. An increase in tear inflammatory cytokines and tear film instability in the early postoperative period was noted; however, this increase was resolved in 6 months in subjects without preexisting dry eye disease^(10)^. Authors suggested that ocular surface inflammation induced by upper blepharoplasty plays an important role in the development of postoperative dry eye and is closely related to subjective symptoms, tear film instability, and meibomian gland dysfunction^(10)^. They also noted that preexisting dry eye was an important risk factor for worse and persistent ocular surface damage after upper eyelid blepharoplasty. In this study, even though we excluded patients with dry eye disease, higher preoperative OSDI scores indicative of higher subjective symptoms might explain our findings^(10)^.

IVCM is a new, noninvasive technology that can help assess structural changes in ocular surface diseases at the cellular level. Many IVCM findings have been reported in dry eye disease^(11,12)^. Studies demonstrated structural alterations in all corneal layers, including sub-basal nerve loss and a reduction in keratocyte density in dry eye disease^(26,27)^. In this study, no significant changes in the corneal microstructure were noted during the study period.

Our study should be interpreted in light of its potential limitations. The major limitation of this study was that the study sample size was relatively limited and that the sample size was not calculated before the study. Our study population mostly included an older patient group where dry eye symptoms are more commonly seen; therefore, our results may not apply to young cosmetic cases. Furthermore, most patients in this study were female, which might have affected our results because dry eye disease is far more prevalent in women^(28,29)^.

Although considered a temporary problem, dry eye symptoms are underestimated and may not resolve with time, particularly in patients with preexisting dry eye complaints. Complete preoperative history and ophthalmic examination should be performed before upper eyelid blepharoplasty. Appropriate preoperative counseling must be provided, particularly for patients with preexisting dry eye complaints.

In conclusion, our study demonstrated deterioration in ocular surface staining, tear film stability, and patient’s subjective symptoms after upper eyelid blepha roplasty with skin and partial preseptal orbicularis removal compared with baseline, without any changes in the corneal microstructure demonstrated by IVCM.
